# Supplementary material for: Expression of Maize MADS Transcription Factor ZmES22 Negatively Modulates Starch Accumulation in Rice Endosperm
Source: Int J Mol Sci. 2019 Jan 23;20(3):483. doi: 10.3390/ijms20030483 (PMC6387075; doi:10.3390/ijms20030483)
Supplement: Supplementary file 1 [file ijms-20-00483-s001.zip › Figure Legends of Supplementary Data.docx]

**Figure S1. Deduced amino acid sequences of *ZmES22*.**

The MADS and K-BOX domains are indicted by solid and dashed lines, respectively.

**Figure S2. Sequence alignment of the conserved MADS domains of genes from maize, rice and *Arabidopsis* MADS family members.**

The locus names of the corresponding genes are as follows: *ZmES22* (GRMZM2G159397), *ZmMADS4* (GRMZM2G032339), *ZmMADS49* (GRMZM2G129034), *OsMADS7* (Os08g0531700), *OsMADS8* (Os09g0507200), *AtAGL2* (AT5G15800), *AtAGL4* (AT3G02310), *AtAGL9* (AT1G24260).

**Figure S3. Transcriptional activation assay of *ZmES22*.**

(A) Schematic diagram of *ZmES22* transcriptional activation construct. PADH1, ADH1 promoter; GAL4 BD, GAL4 DNA-binding domain; PT7, T7 promoter; TT7&ADH1, T7 and ADH1 transcription termination signals. (B) The pGBKT7-*ZmES22* construct, containing HIS3, ADE2 and MEL1 reporter genes, was transformed into yeast strain AH109. Positive control, the co-transformants of pGBKT7-53 and pGADT7-T; negative control, the transformants of pGBKT7. The results were examined on SD/-Trp and SD/-Trp/-His/-Ade/X-α-GAL medium for 2–3 d at 30°C.

**Figure S4. mRNA expression level of *ZmES22* gene in wide type (Zhonghua 11) and three independent transgenic lines of *ZmES22* via quantitative RT-PCR.**

L8, L9 and L10 were three distinct transgenic rice lines that overexpressed *ZmES22*. *OsTubulin* expression was used as an internal control. Data are shown as the mean ± SD of three biological and two technical replicates.

**Figure S5. Phenotypes of transgenic rice plants and WT plants.**

(A) The left panel represents phenotypes of wide type (Zhonghua 11) and three independent transgenic lines of *ZmES22* (L8, L9 and L10) at heading stage under natural LD (NLD) conditions in Hefei city, China. (B) the right panel denotes rice tassel of wide type (Zhonghua 11) and three independent transgenic lines of *ZmES22* (L8, L9 and L10).

**Figure S6. GO enrichment analysis of DEGs between wild type and transgenic rice lines.**

Gene enrichment analysis was complemented for DEGs between wild type plants and transgenic rice lines that overexpressed *ZmES22*. The black circle represented number of genes that were enriched in GO item, the color panel denoted *P*-value for each GO item.

**Figure S7. Starch and sucrose metabolism pathway that was significantly enriched for DEGs between wild type and transgenic rice lines.**

**Figure S8. Plant hormone signal transduction pathway that was significantly enriched for DEGs between wild type and transgenic rice lines.**

**Figure S9. Expression profiles of *OsGIF1* during seed development in wild-type Zhonghua11 and three independent transgenic lines of *ZmES22* (L8, L9 and L10).**

Total RNA was extracted from seeds at 3, 6, 10, 20 DAP, respectively. The mRNA expression level of *OsGIF1* in the 3 DAP seeds of Zhonghua11 was used to normalize the dataset.
