# Supplementary material for: Expression of Maize MADS Transcription Factor ZmES22 Negatively Modulates Starch Accumulation in Rice Endosperm
Source: Int J Mol Sci. 2019 Jan 23;20(3):483. doi: 10.3390/ijms20030483 (PMC6387075; doi:10.3390/ijms20030483)
Supplement: Supplementary file 1 [file ijms-20-00483-s001.zip › Table of Supplementary Data.docx]

| **Table S1. Basic information of the 16 homeodomains of some maize, rice and Arabidopsis MADS family members.** | | | | | | |
| --- | --- | --- | --- | --- | --- | --- |
| Sample name | Gene IDs | Chromosome | Start | End | Strand | Predicted functions |
| *ZmMADS14* | GRMZM2G099522 | Chr1 | 26671645 | 26681203 | - | MADS-box transcription factor 1-like |
| *ZmMADS8* | GRMZM2G102161 | Chr9 | 146936987 | 146945281 | + | MADS-box transcription factor 1-like |
| *ZmMADS31* | GRMZM2G071620 | Chr5 | 6925447 | 6932461 | + | MADS-box transcription factor 1-like |
| *ZmMADS24* | GRMZM2G087095 | Chr1 | 277377468 | 277415566 | - | MADS-box transcription factor 1-like |
| *ZmES22* | GRMZM2G159397 | Chr1 | 194046502 | 194054556 | + | MADS-box transcription factor 1-like |
| *ZmMADS4* | GRMZM2G032339 | Chr1 | 277218151 | 277298925 | - | MADS-box transcription factor 1-like |
| *ZmMADS49* | GRMZM2G129034 | Chr2 | 192877045 | 192885419 | + | MADS-box transcription factor 1-like |
| *OsMADS1* | Os03g0215400 | Chr3 | 6052750 | 6061369 | - | Involved in flower induction |
| *OsMADS5* | Os06g0162800 | Chr6 | 3162801 | 3169415 | - | Regulation of reproductive development |
| *OsMADS34* | Os03g0753100 | Chr3 | 31048351 | 31055019 | - | Inflorescence branching,grain number, female sterility |
| *OsMADS7* | Os08g0531700 | Chr8 | 26507180 | 26512256 | + | Regulation of reproductive development |
| *OsMADS8* | Os09g0507200 | Chr9 | 19653389 | 19659766 | + | Regulation of reproductive development |
| *AtAGL3* | AT2G03710 | Chr2 | 1129268 | 1131838 | + | Plays a central role in the determination of flower meristem and organ identity |
| *AtAGL4* | AT3G02310 | Chr3 | 464279 | 467074 | - | MADS-box protein, binds K domain of AG in vivo |
| *AtAGL2* | AT5G15800 | Chr5 | 5151334 | 5154154 | - | Encodes a MADS box transcription factor involved flower and ovule development |
| *AtAGL9* | AT1G24260 | Chr1 | 8593642 | 8595909 | - | SEP3 forms heterotetrameric complexes with other MADS box family members |

| **Table S2. The degree of difference between the 16 homeodomains of some maize, rice and Arabidopsis MADS family members.** | | | | | | | | | | | | | | | | |
| --- | --- | --- | --- | --- | --- | --- | --- | --- | --- | --- | --- | --- | --- | --- | --- | --- |
| Sample name | *ZmMADS14* | *ZmMADS8* | *OsMADS1* | *OsMADS5* | *OsMADS34* | *ZmMADS31* | *ZmMADS24* | *AtAGL3* | *AtAGL4* | *AtAGL2* | *AtAGL9* | *OsMADS7* | *ZmES22* | *OsMADS8* | *ZmMADS4* | *ZmMADS49* |
| *ZmMADS14* |  |  |  |  |  |  |  |  |  |  |  |  |  |  |  |  |
| *ZmMADS8* | 0.14 |  |  |  |  |  |  |  |  |  |  |  |  |  |  |  |
| *OsMADS1* | 0.26 | 0.24 |  |  |  |  |  |  |  |  |  |  |  |  |  |  |
| *OsMADS5* | 0.37 | 0.34 | 0.29 |  |  |  |  |  |  |  |  |  |  |  |  |  |
| *OsMADS34* | 0.78 | 0.75 | 0.67 | 0.7 |  |  |  |  |  |  |  |  |  |  |  |  |
| *ZmMADS31* | 0.74 | 0.73 | 0.65 | 0.71 | 0.22 |  |  |  |  |  |  |  |  |  |  |  |
| *ZmMADS24* | 0.72 | 0.72 | 0.65 | 0.71 | 0.22 | 0.05 |  |  |  |  |  |  |  |  |  |  |
| *AtAGL3* | 0.85 | 0.84 | 0.79 | 0.83 | 0.78 | 0.71 | 0.71 |  |  |  |  |  |  |  |  |  |
| *AtAGL4* | 0.79 | 0.78 | 0.73 | 0.7 | 0.74 | 0.75 | 0.72 | 0.71 |  |  |  |  |  |  |  |  |
| *AtAGL2* | 0.75 | 0.73 | 0.73 | 0.69 | 0.75 | 0.72 | 0.68 | 0.64 | 0.16 |  |  |  |  |  |  |  |
| *AtAGL9* | 0.79 | 0.79 | 0.74 | 0.76 | 0.79 | 0.8 | 0.76 | 0.75 | 0.55 | 0.5 |  |  |  |  |  |  |
| *OsMADS7* | 0.8 | 0.8 | 0.78 | 0.72 | 0.79 | 0.77 | 0.73 | 0.73 | 0.56 | 0.57 | 0.54 |  |  |  |  |  |
| *ZmES22* | 0.81 | 0.79 | 0.75 | 0.68 | 0.79 | 0.78 | 0.72 | 0.68 | 0.57 | 0.53 | 0.51 | 0.19 |  |  |  |  |
| *OsMADS8* | 0.76 | 0.77 | 0.75 | 0.71 | 0.81 | 0.75 | 0.71 | 0.7 | 0.55 | 0.55 | 0.52 | 0.26 | 0.28 |  |  |  |
| *ZmMADS4* | 0.77 | 0.73 | 0.7 | 0.72 | 0.78 | 0.72 | 0.68 | 0.71 | 0.58 | 0.56 | 0.56 | 0.31 | 0.33 | 0.14 |  |  |
| *ZmMADS49* | 0.75 | 0.71 | 0.68 | 0.68 | 0.77 | 0.72 | 0.69 | 0.71 | 0.55 | 0.53 | 0.52 | 0.27 | 0.3 | 0.09 | 0.06 |  |

| **Table S3. Primers used in this study** | | |
| --- | --- | --- |
| **Use** | **Primer name** | **Sequence (5’ to 3’)** |
| qRT-PCR for starch synthesis genes | ***OsTubulin-F*** | TACCGTGCCCTTACTGTTCC |
|  | ***OsTubulin-R*** | CGGTGGAATGTCACAGACAC |
|  | ***OsAGPL1-F*** | GGAAGACGGATGATCGAGAAAG |
|  | ***OsAGPL1-R*** | CACATGAGATGCACCAACGA |
|  | ***OsAGPL2-F*** | AGTTCGATTCAAGACGGATAGC |
|  | ***OsAGPL2-R*** | CGACTTCCACAGGCAGCTTATT |
|  | ***OsAGPS1-F*** | GTGCCACTTAAAGGCACCATT |
|  | ***OsAGPS1-R*** | CCCACATTTCAGACACGGTTT |
|  | ***OsAGPS2b-F*** | AACAATCGAAGCGCGAGAAA |
|  | ***OsAGPS2b-R*** | GCCTGTAGTTGGCACCCAGA |
|  | ***OsBEI-F*** | TGGCCATGGAAGAGTTGGC |
|  | ***OsBEI-R*** | CAGAAGCAACTGCTCCACC |
|  | ***OsBEIIa-F*** | GCCAATGCCAGGAAGATGA |
|  | ***OsBEIIa-R*** | GCGCAACATAGGATGGGTTT |
|  | ***OsBEIIb-F*** | ATGCTAGAGTTTGACCGC |
|  | ***OsBEIIb-R*** | AGTGTGATGGATCCTGCC |
|  | ***OsGBSSI-F*** | AACGTGGCTGCTCCTTGAA |
|  | ***OsGBSSI-R*** | TTGGCAATAAGCCACACACA |
|  | ***OsGBSSII-F*** | AGGCATCGAGGGTGAGGAG |
|  | ***OsGBSSII-R*** | CCATCTGGCCCACATCTCTA |
|  | ***OsISA1-F*** | TGCTCAGCTACTCCTCCATCATC |
|  | ***OsISA1-R*** | AGGACCGCACAACTTCAACATA |
|  | ***OsISA2-F*** | TAGAGGTCCTCTTGGAGG |
|  | ***OsISA2-R*** | AATCAGCTTCTGAGTCACCG |
|  | ***OsISA3-F*** | ACAGCTTGAGACACTGGGTTGAG |
|  | ***OsISA3-R*** | GCATCAAGAGGACAACCATCTG |
|  | ***OsPUL-F*** | ACCTTTCTTCCATGCTGG |
|  | ***OsPUL-R*** | CAAAGGTCTGAAAGATGGG |
|  | ***OsSSI-F*** | GGGCCTTCATGGATCAACC |
|  | ***OsSSI-R*** | CCGCTTCAAGCATCCTCATC |
|  | ***OsSSIIa-F*** | GCTTCCGGTTTGTGTGTTCA |
|  | ***OsSSIIa-R*** | CTTAATACTCCCTCAACTCCACCAT |
|  | ***OsSSIIIa-F*** | GCCTGCCCTGGACTACATTG |
|  | ***OsSSIIIa-R*** | GCAAACATATGTACACGGTTCTGG |
|  | ***OsSSIVa-F*** | GGGAGCGGCTCAAACATAAA |
|  | ***OsSSIVa-R*** | CCGTGCACTGACTGCAAAAT |
| qRT-PCR forDEGs | ***OsTubulin-F*** | TACCGTGCCCTTACTGTTCC |
|  | ***OsTubulin-R*** | CGGTGGAATGTCACAGACAC |
|  | ***OS01G0638000-F*** | ACGAGAAGCTCATCACGGAG |
|  | ***OS01G0638000-R*** | TCGCCCATCACTCTCCCTAT |
|  | ***OS08G0535200-F*** | GAGGTTTCTTGTCCATGGCTAAC |
|  | ***OS08G0535200-R*** | CGTCGACTTCTTCTTGTACACCT |
|  | ***OS01G0597800-F*** | AATGATCGACCCAAAGAAACGG |
|  | ***OS01G0597800-R*** | CCCACATTTCAGACACGGTTT |
|  | ***OS03G0687700-F*** | ATCAGGGAGACGGTGTCGA |
|  | ***OS03G0687700-R*** | ACCTCGAACGACATGGTGTC |
|  | ***OS11G0454000-F*** | GATGGGAATGGGAGGGCATC |
|  | ***OS11G0454000-R*** | CTTCTTCCTCCTCCCTCCCA |
|  | ***OS04G0526800-F*** | GTCCATGTCATCGGAGTCCC |
|  | ***OS04G0526800-R*** | AGACGTATAAGGGCTGGGGT |
|  | ***OS09G0417800-F*** | CTTACTTCCGCTGCGCATTC |
|  | ***OS09G0417800-R*** | GACGAATTCGGTTGTCTGCG |
|  | ***OS05G0331532-F*** | GTGGCGCAACAGTCTCACTA |
|  | ***OS05G0331532-R*** | AGCTTGAGCCAGATTCCGAC |
|  | ***OS11G0454200-F*** | AGCAGCAGATGATGGGGAAC |
|  | ***OS11G0454200-R*** | TGAATCCCTTCTTCTCGCCG |
|  | ***OS12G0269100-F*** | CAGCACAGTGGAAATCCCCT |
|  | ***OS12G0269100-R*** | GCTGTAGCTGCTGCACAATC |
