# Supplementary figures and images for: Expression of Maize MADS Transcription Factor ZmES22 Negatively Modulates Starch Accumulation in Rice Endosperm
Source: Int J Mol Sci. 2019 Jan 23;20(3):483. doi: 10.3390/ijms20030483 (PMC6387075; doi:10.3390/ijms20030483)

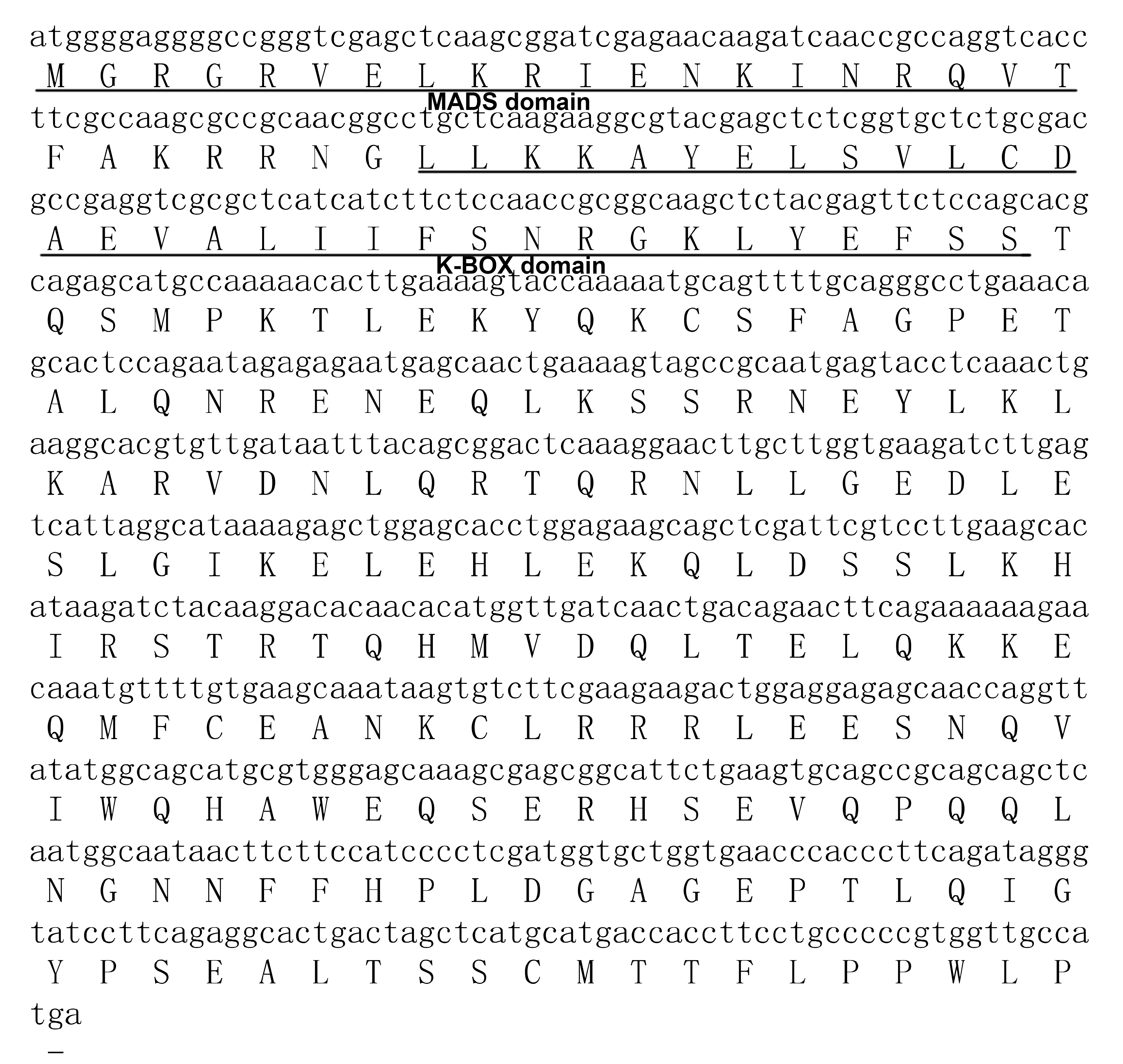

Supplement: Supplementary file 1 [file ijms-20-00483-s001.zip › Fig. S1.tif]

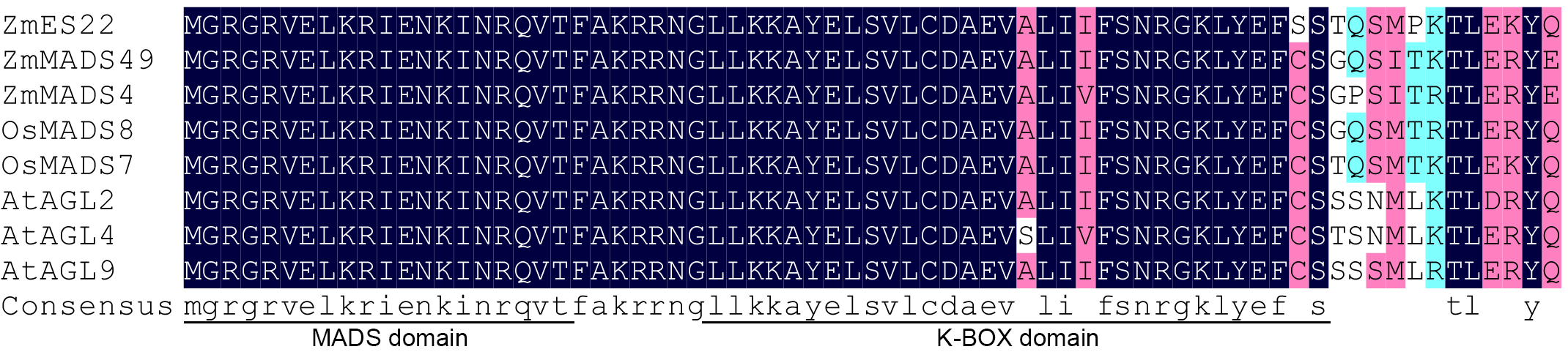

Supplement: Supplementary file 1 [file ijms-20-00483-s001.zip › Fig. S2.tif]

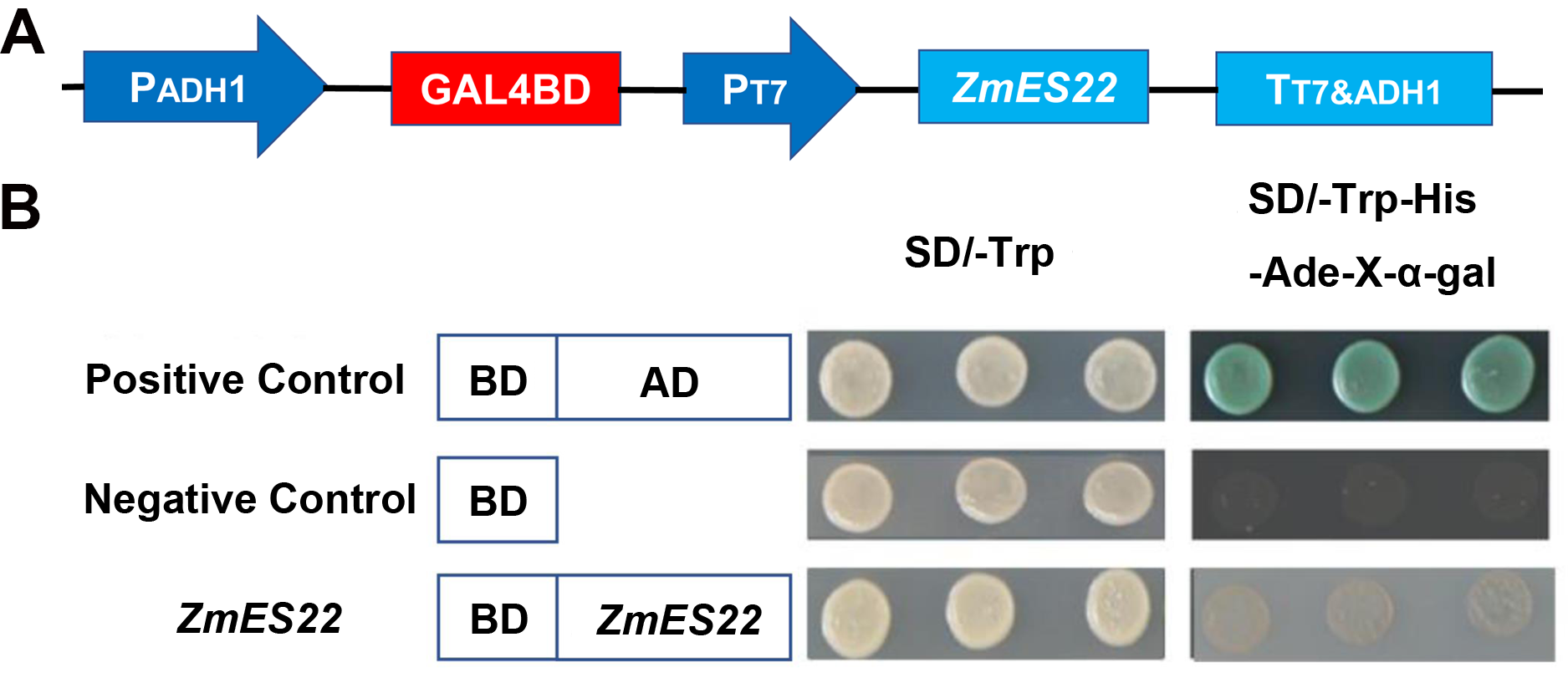

Supplement: Supplementary file 1 [file ijms-20-00483-s001.zip › Fig. S3.tif]

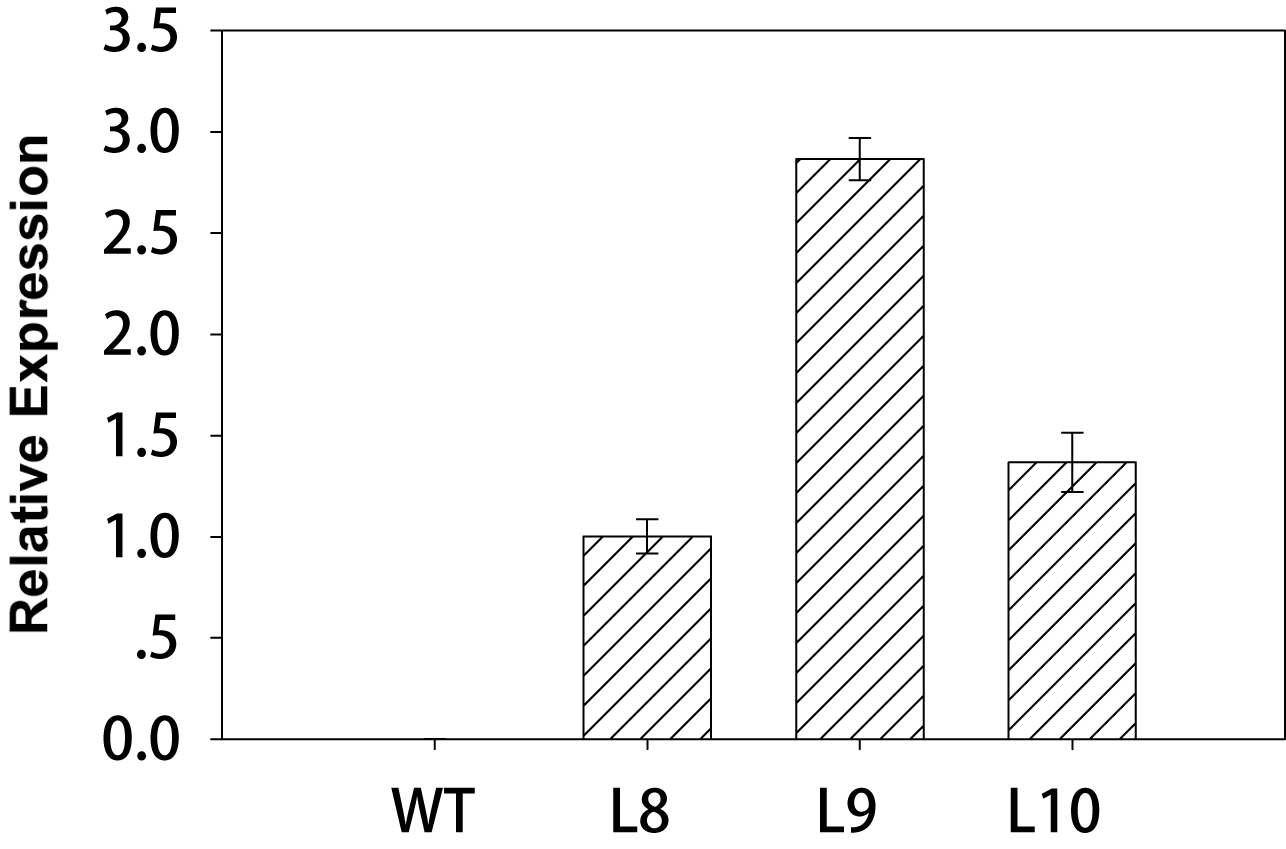

Supplement: Supplementary file 1 [file ijms-20-00483-s001.zip › Fig. S4.tif]

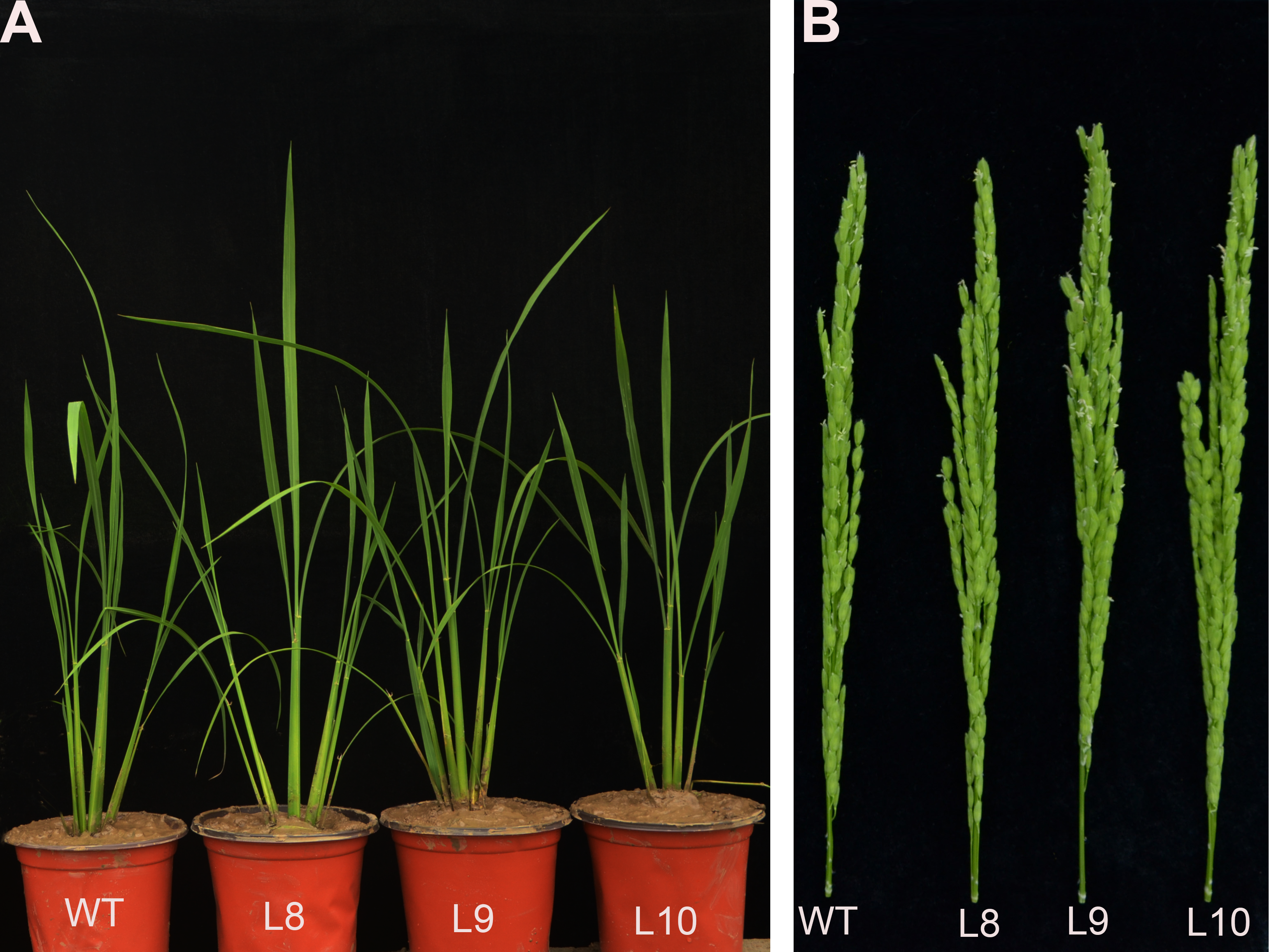

Supplement: Supplementary file 1 [file ijms-20-00483-s001.zip › Fig. S5.tif]

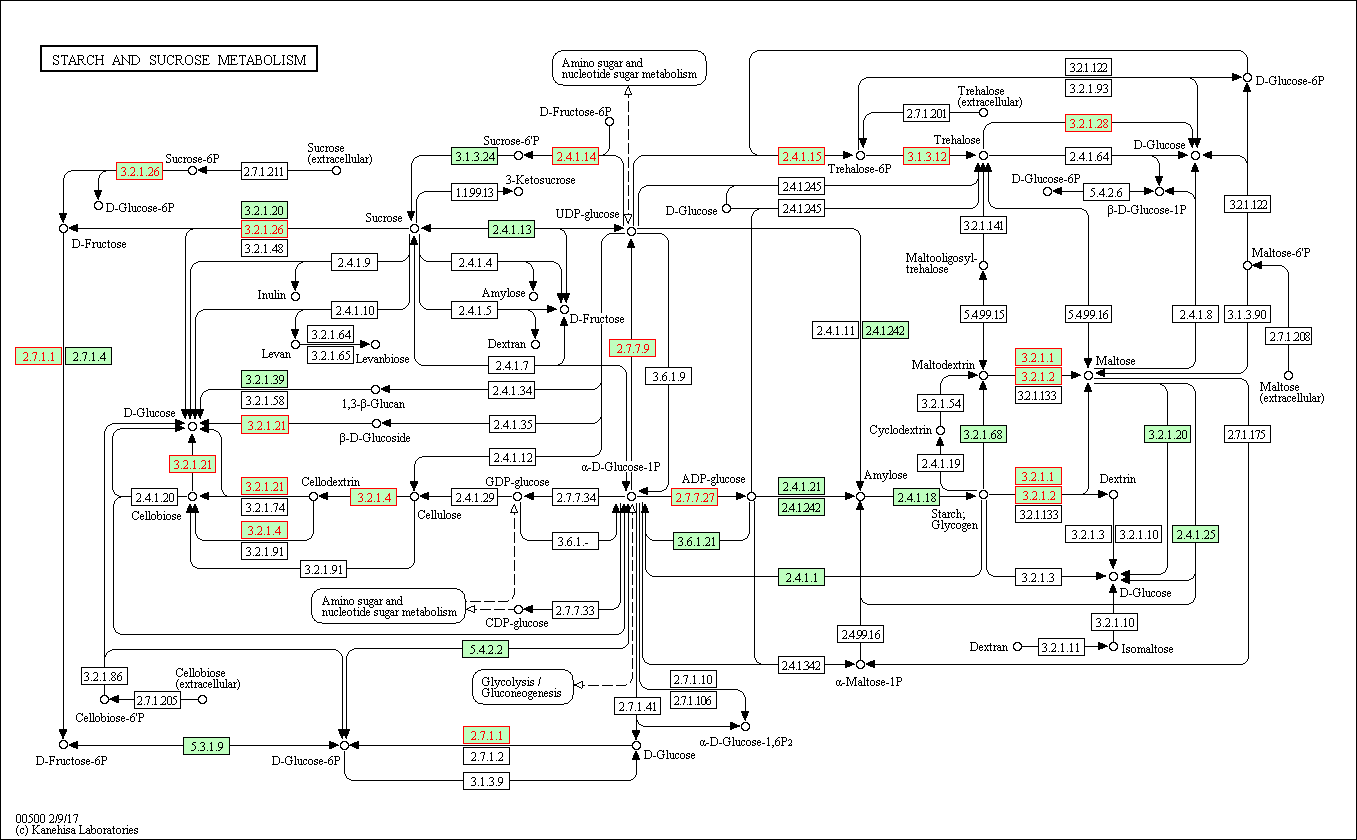

Supplement: Supplementary file 1 [file ijms-20-00483-s001.zip › Fig. S7.tif]

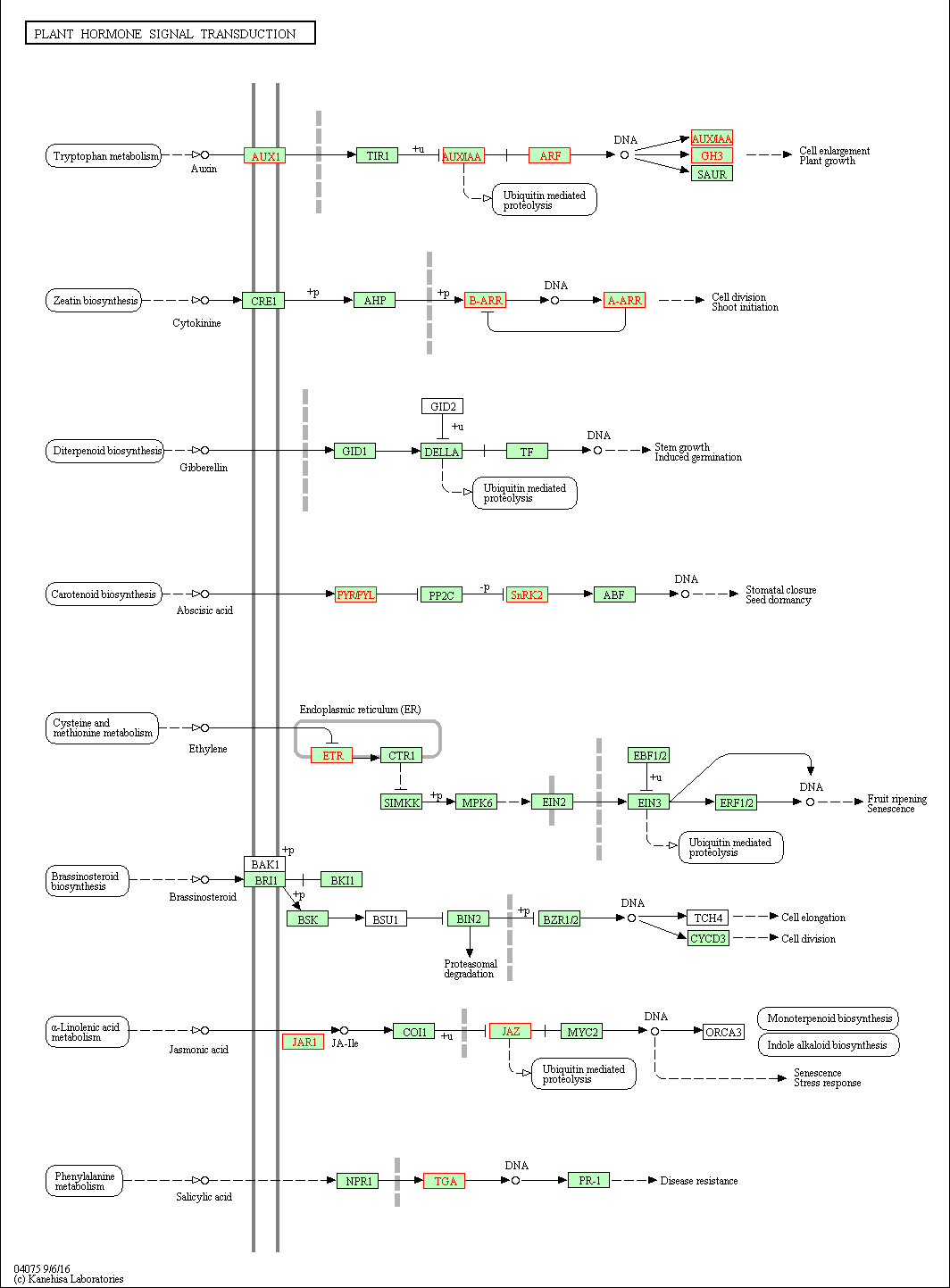

Supplement: Supplementary file 1 [file ijms-20-00483-s001.zip › Fig. S8.tif]

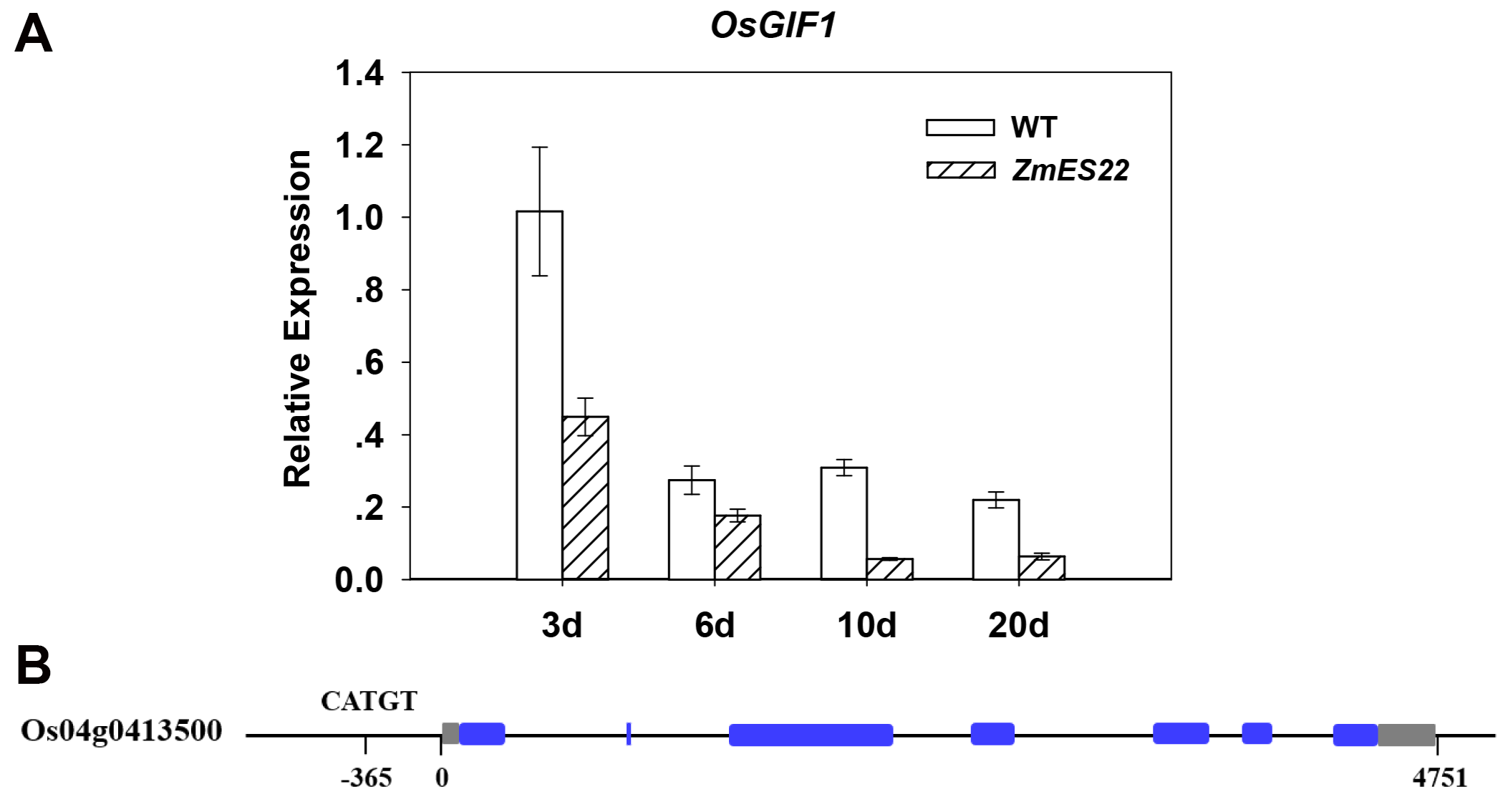

Supplement: Supplementary file 1 [file ijms-20-00483-s001.zip › Fig. S9.tif]
